# Ginsenoside Rk3 Inhibits the Extramedullary Infiltration of Acute Monocytic Leukemia Cell via miR-3677-5p/CXCL12 Axis

**DOI:** 10.1155/2022/3065464

**Published:** 2022-12-16

**Authors:** Shanshan Ma, Qingcheng Huang, Qinxue Hu, Ruilan Gao, Jinjian Lan, Xiaoling Yu, Yanna Zhao, Fenglin Shen, Ai Mi, Bolin Wang

**Affiliations:** ^1^Institute of Hematology Research, The First Affiliated Hospital of Zhejiang Chinese Medical University, Hangzhou, Zhejiang, China; ^2^Zhejiang Provincial Hospital of Chinese Medicine, Hangzhou, Zhejiang, China

## Abstract

**Background:**

Acute monocytic leukemia belongs to type M5 of acute myeloid leukemia (AML) classified by FAB, which appears a high incidence of extramedullary infiltration (EMI) and poor prognosis. In this study, we observed the inhibitory effect of ginsenoside Rk3 on the EMI of monocytic leukemia cells and initially explored its related mechanism of targeting the miR-3677-5p/CXCL12 axis.

**Methods:**

The MTT assay and colony formation assay were used to detect the inhibitory effect of Rk3 on proliferation. Both cellular migration and invasion were observed by the Transwell assay. The expression levels of miR-3677-5p, CXCL12, and CXCR4 were detected by RT-qPCR and Western blot, as well as overexpression of miR-3677-5p by transfected with lentivirus and detection of a dual luciferase reporter gene. The expression of MMP2 and TIMP2 was detected by immunofluorescence.

**Results:**

Rk3 effectively inhibits the proliferation, migration, and invasion associated with EMI of leukemia. The leukemia cells of M5 patients with EMI showed low expression of miR-3677-5p but high expression of the mRNA of CXCL12 and CXCR4. Overexpression of miR-3677-5p or intervention of CXCL12 effectively inhibited the proliferation, migration, and invasion of SHI-1 cells. The luciferase assay showed that CXCL12 was the downstream target gene of miR-3677-5p. After overexpression of miR-3677-5p or intervention of CXCL12 in combination with Rk3, the inhibitory effect on the proliferation, migration, and invasion of SHI-1 cells was more obvious. Importantly, Rk3 significantly regulated the expression levels of miR-3677-5p, CXCL12, CXCR4, and EMI-related functional proteins including MMP2 and TIMP2. Overexpression of miR-3677-5p or intervention of CXCL12 also regulated the expression of MMP2 and TIMP2.

**Conclusions:**

The leukemia cells of M5 patients with EMI appeared to have low expression of miR-3677-5p and high expression of the mRNA of CXCL12 and CXCR4, which may be used as indicators of EMI and poor prognosis. Rk3 is effective in inhibiting the EMI of SHI-1 cells by targeting the miR-3677-5p/CXCL12 axis.

## 1. Introduction

Acute myeloid leukemia (AML) is a clonal, malignant disease of the hematopoietic tissues that is characterized by the accumulation of abnormal leukemic blast cells, principally in the marrow, and impaired production of normal blood cells. Thus, the leukemic cell infiltration in the marrow is accompanied, nearly invariably, by anemia and thrombocytopenia. The absolute neutrophil count may be low or normal, depending on the total white cell count [1]. Acute monocytic leukemia belongs to type M5 of AML, as classified by FAB. The incidence of extramedullary infiltration (EMI) in clinical patients with M5 leukemia is as high as 30% to 40%. Infiltration of the central nervous system is a serious complication, leading to recurrence and death, with a poor prognosis and no specific effective medicine. The treatment of patients is still dominated by chemotherapy, and the main problems of chemotherapy are treatment-related toxicity, mortality, and the occurrence of multidrug resistance (MDR) [2–4]. Existing targeted therapies and immunotherapy is also not ideal for prevention and radical cure of EMI. Furthermore, they are expensive. Therefore, it is of important clinical value to research and develop new Chinese medicine or natural compounds that are safe and effective for anti-EMI so as to improve the prognosis of M5 leukemia patients.

Ginseng is a Chinese medicine herb, and its main ingredient is ginsenosides, which are characteristics of anti-inflammatory properties, antitumor and other pharmacological effects [5–9]. Our previous work has identified a biological active compound extracted and isolated from ginseng herb by using biological activities assay as indicators, we termed the purified polarity ginsenosides as ALK compound, the invention patent was authorized by Patent Office of China, which possesses clear effects on inhibiting the proliferation and inducing differentiation in AML cells, as well as anti-EMI of central nervous system and other organs, which caused by monocytic leukemia SHI-1 cells in mice models with EMI. In order to explore the material basis of the ALK compound for antileukemia, the purified polarity ginsenoside monomers of F4, Rh4, and Rk3 were isolated from ALK, and the results showed that F4 significantly inhibited the proliferation, and also induced the differentiation of K562 and SHI-1 leukemia cells [10]. Ginsenoside Rh4 was effective to inhibit the proliferation, arrest cell cycle, and induce differentiation of K562 cells [11]. Since Rk3, nothing has been reported about its action and mechanism in antileukemia.

MicroRNA (miRNA) is a single-stranded RNA molecule of about 22 nucleotides, located at the core of the ncRNA regulatory network. Its function is to participate in the maintenance of cellular proliferation, differentiation, and apoptosis. Abnormal expression or regulation of miRNAs is closely related to the occurrence and progression of leukemia/tumor [12–16]. Studies have shown that miR-3677 is low expressed in serum samples of osteosarcoma patients and is associated with a poor prognosis of osteosarcoma [17]. Overexpression of miR-3677 can inhibit the proliferation and migration of osteosarcoma cells [18]. But the expression level of miR-3677 and its specific mechanism of actin have not yet been elucidated in acute monocytic leukemia. The miR-3677-5p is processed from the 5′ end arm of the miR-3677 precursor. CXCL12 is a ligand of the G-protein-coupled receptor CXCR4, and CXCR4 and its ligand-induced signal cascade are usually defined as the CXCL12/CXCR4 axis [19]. The CXCL12/CXCR4 axis is involved in the survival and homing of hematopoietic stem cells in bone marrow and is closely related to the proliferation and invasion of malignant cells [20–22].

Therefore, we studied the effects of miR-3677-5p and CXCL12 expression on the proliferation, migration, and invasion of SHI-1 cells and explored the active mechanism of Rk3 for anti-EMI in monocytic leukemia cells.

## 2. Materials and Methods

### 2.1. Cell Culture, Lentivirus Transfection, and Treatment

Human monocytic leukemia SHI-1 cells were donated by Professor Chen Zixing, Institute of Hematology, and the First Affiliated Hospital of SooChow University. The cells were maintained in Iscove's Modified Dulbecco's Medium (IMDM, Gibco, USA) supplemented with 15% new bovine serum (NBS, Every Green, China) in a humidified 5% CO_2_ atmosphere at 37°C. The structure of ginsenoside Rk3 (WINHERB, Shanghai, China) was identified by nuclear magnetic resonance, mass, infrared spectrum, and ultraviolet absorption spectra. The purity was >98%, and the batch No. 140606. Polybrene transfection reagent was used for the transfection of miR-NC (GenePharma, Shanghai, China) and miR-3677-5p mimic (GenePharma, Shanghai, China) according to the manufacturer's instructions. The AMD3100 (the inhibitor of the CXCL12/CXCR4 axis) was purchased from Selleck Chemicals (Shanghai, China), and the batch No. S803002.

### 2.2. Collection of Bone Marrow Specimens and Clinical Data

The bone marrow samples from 20 patients diagnosed with AML-M5 were collected in our hospital from April 2021 to March 2022; the diagnostic criteria were based on the 2016 World Health Organization Classification of AML [23]. The bone marrow samples were aspirated before chemotherapy, and anticoagulated by heparin, afterwards, leukemia cells were isolated from these bone marrow samples. The patient clinical manifestation and data were recorded, including extramedullary infiltration (EMI) or nonextramedullary infiltration (N-EMI), the percentage of bone marrow leukemia blasts, chemotherapy regimens, and response to chemotherapy (Table 1). 20 cases of AML patients were divided into the EMI group (10 cases) and the N-EMI group (10 cases) according to their clinical manifestations. Patients in the EMI group showed infiltration of brain tissue (according to the cerebrospinal fluid examination), enlargement of the liver, spleen, lymph nodes, and tenderness of the sternum, which suggested that leukemia cells were infiltrated in these organs. The normal control group consisted of 10 normal bone marrow samples, which required bone marrow examination for clinical diagnosis, but the bone marrow smears showed normal hematopoietic morphology. This study was approved by the Ethics Committee of the First Affiliated Hospital of Zhejiang Chinese Medicine University.

### 2.3. MTT Assay

About 2 × 10^4^ SHI-1 cells were plated into each well of a 96-well plate, and treated with Rk3 at the final concentration of 60, 120, and 180 mol/L for 48 hours at 37°C in a 5% CO_2_ incubator, and each group of Rk3 concentration was repeated planting for 5 wells. Then, 20 *μ*l MTT solutions (5 mg/mL) was added and incubated for 4 hours. After the supernatant was removed by centrifugation, 150 *μ*l DMSO was added, the absorbance of the solution was read by a microplate reader ELx808 (BioTek, USA), using a test wavelength of 490 nm. The inhibition rate of Rk3 on the proliferation of SHI-1 cells was calculated, the inhibition rate (%) = [(control group A value − experimental group A value)/control group A value] × 100%. The same experiment was repeated for 5 times.

### 2.4. Semisolid Culture for Colony Forming Assay

SHI-1 cells were cultured in IMDM consisting of 30% new bovine serum, 1% L-glutamine, penicillin-streptomycin, and 0.3% agar as viscous support, with the final concentrations of Rk3 were 60, 120, and 180 mol/L. In addition, the other three groups, including miR-3677-5p mimic, miR-NC, and 2.5 *μ*M of AMD3100, were treated for 12 hours. They were planted to the semisolid culture system by the method above. The experiments were performed in triplicate with 10^3^ cells per well in a 24-well plate, and incubated at 37°C in a humidified atmosphere supplemented with 5% CO_2_ for 7 days. The colony number (≥40 cells) was counted under the microscope. The inhibition rate of Rk3/miR-3677-5p mimic/AND3100 on proliferation of SHI-1 cells (%) = [(colony number in control group − colony number in experimental group)/colony number in control group] × 100%. The same experiment was repeated for 5 times.

### 2.5. Transwell Assay

SHI-1 cells were treated with Rk3 at a final concentration of 60, 120, and 180 mol/L for 48 hours. In addition, the other three groups included miR-3677-5p mimic, miR-NC, and 2.5 *μ*M of AMD3100, which were collected for the Transwell assay. In the migration experiment, 1 × 10^5^ cells (200 *μ*l) were seeded into the upper chamber of a Transwell insert (8 mm pore size, Corning Inc., USA), and a medium with 15% NBS (600 *μ*l) was added in the lower chamber. In the invasion experiment, Matrigel (BD Bioscience, Shanghai, China) was coated on the upper chamber seeded with 1 × 10^5^ cells, and the lower chamber contained 15% NBS medium. After incubation at 37°C, 5% CO_2_ for 24 hours, the Transwell chamber was taken out and washed with PBS. The cells on the membrane were fixed with 4% paraformaldehyde for 20 mins, and stained with crystal violet for 30 mins. The upper unmigrated cells were gently wiped off with a cotton swab, and five fields were randomly selected under the microscope (100 × magnification, Olympus, Japan) to take photos and count cell numbers.

### 2.6. Bioinformatics Analysis

The Gene Expression Profiling Interactive Analysis (GEPIA) website (https://gepia.cancer-pku.cn/detail.php###) was used to analyze the difference in CXCL12 and CXCR4 expression levels between the AML patients group (num = 173) and the normal control group (num = 70).

### 2.7. Quantitative Real-Time PCR

SHI-1 cells were incubated with 60, 120, and 180 mol/L of Rk3 for 48 hours, in addition, the other three groups, including miR-3677-5p mimic, miR-NC, and 2.5 *μ*M of AMD3100, which were washed with ice-cold PBS, and total RNA was extracted by TRIzol. The cDNAs were synthesized from total RNA using the RT kit (Thermo Fisher Scientific, Lithuania). PowerUp™ SYBR Green Master Mix (Thermo Fisher Scientific, Lithuania) was applied to the thermocycling reaction in the QuantStudio 7 Flex real-time PCR system. The forward and reverse primers of *β*-actin were 5′-CATCAAGGAGAAACTGTGT-3′ and 5′-AGGCAACTCGTAACTCTT-3′; the forward and reverse primers of U6 were 5′-AGAGAAGATTAGCATGGCCCCTG-3′ and 5′-CAGTGCAGGGTCCGAGGT-3′, the forward and reverse primers of miR-3677-5p were 5′-GCAGTGGCCAGAGCCCT-3′ and 5′-AGTGCAGGGTCCGAGGTATT-3′, the forward and reverse primers of CXCL12 were 5′-ATTCATAACACTCCAAACTGTGC-3′ and 5′-ACTTTAGCTTCGGGTCAATGC-3′, the forward and reverse primers of CXCR4 were 5′-ACTACACCGAGGAAATGGGCT-3′ and 5′-CCCACAATGCCAGTTAAGAAGA-3′. The thermal cycler conditions were performed as follows: Cycle 1 (95°C for 3 mins), Cycle 2 (35 cycles of 95°C for 30 s, 57°C for 30 s, and 72°C for 30 s), and Cycle 3 (72°C for 4 mins). The relative gene expression quantification was calculated with the 2^−ΔΔCt^ method. All experiments were done in triplicate. The RNAs of leukemia cells from patients with M5 were handled by the same method. The same experiment was repeated for 3 times. *β*-actin was used as an internal control.

### 2.8. Western Blot

Cells were washed twice with ice PBS, and total cell lysate samples were extracted and subjected to SDS-PAGE gel, then, transferred to a nitrocellulose membrane (Amersham, USA), and blocked with Protein-Free Rapid Blocking Buffer (Epizyme, China). The membranes were then reacted with primary antibodies including CXCL12 (Abcam9797, 1 : 1000), CXCR4 (SC53534, 1 : 1000), and *β*-actin (CST13E5, 1 : 1000) overnight at 4°C. After being washed with 1 × TBST buffer for three times, the membranes were incubated with secondary antibody (BIO-RAD, 1 : 3000, USA) for 1 hour at room temperature. The special bands from the conjugation reaction of protein antigen and antibodies were visualized by using Luminol from the enhanced chemiluminescence (ECL) kit (Santa Cruz, USA). Specific protein bands were measured with the densitometric intensity (SHST, China). The experiment above was repeated 3 times.

### 2.9. The Assay of Dual Luciferase Reporter Gene

The binding sites of miR-3677-5p and CXCL12 were predicted using a biological information database (https://www.targetscan.org). Wild-type GP-miRglo-CXCL12 (CXCL12-3′ UTR-Wt) and mutant-type GP-miRglo-CXCL12 (CXCL12-3′ UTR-Mut) dual luciferase reporter plasmids were designed and synthesized based on binding sites. The miR-3677-5p mimic/NC mimic and CXCL12-3′ UTR-Wt/CXCL12-3′ UTR-Mut were transfected into 293T cells using GP-transfected transfection reagent. The luciferase activity was detected after a 48 h reaction. The calculation formula of the relative fluorescence value was = firefly luciferase fluorescence value/ranilla luciferase fluorescence value. The same experiment was repeated for three times.

### 2.10. Immunofluorescence

SHI-1 cells were collected after being treated with 60, 120, and 180 mol/L of Rk3 for 48 hours,and the other groups included miR-3677-5p mimic, miR-NC, and 2.5 *μ*M of AMD3100. Then, prepared smear slides by cyto-centrifugation at 800 rpm for 5 mins. The slides were fixed for 10 mins in 4% paraformaldehyde and treated with 0.5% TritonX-100 for 20 mins. Nonspecific binding was blocked by incubating with 1% BSA. The primary antibodies, including MMP2 (CST40994, 1 : 200) and TIMP2 (Abcam180630, 1 : 200) were incubated over night at 4°C, and secondary antibodies were incubated for 1 hour in the dark. The nuclei were counterstained with DAPI. The cellular fluorescence intensity on slides was observed under the fluorescence microscope of ZEISS. The fluorescence intensity was calculated using Image J software.

### 2.11. Statistical Methods

The data were analyzed using GraphPad Prism software (La Jolla, CA, USA) and shown as mean ± Standard error of the mean. The data comparison was conducted by using the Student's *t*-test or One-way ANOVA, and *P* < 0.05 was considered as statistically significant.

## 3. Results

### 3.1. Rk3 Inhibited the Proliferation of SHI-1 Cells

**Figure 1:**
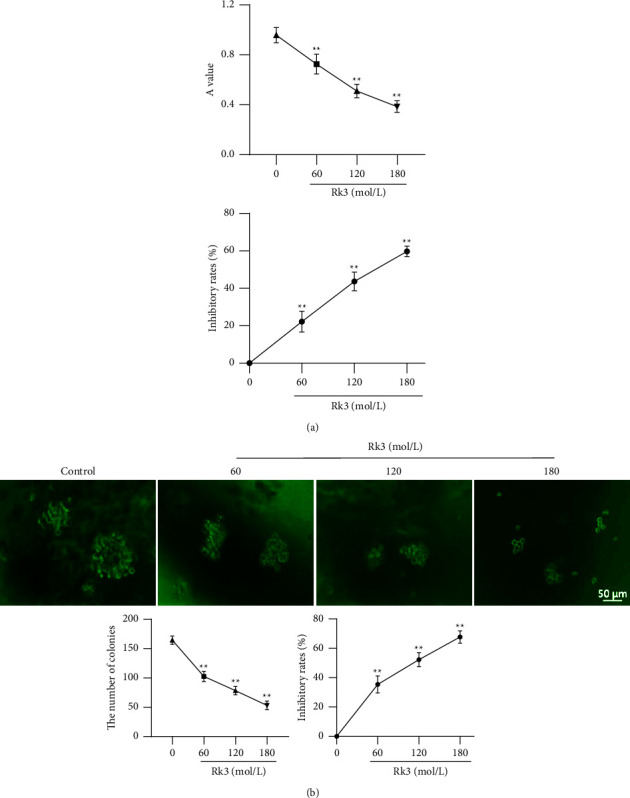
Rk3 effectively inhibited the proliferation in SHI-1 cells. ($\bar{x}\pm s$, *n* = 5) (a) The MTT absorbance value of SHI-1 cells treated by Rk3 was decreased significantly in a dose-dependent manner. (b) The colony numbers of SHI-1 cells treated by Rk3 was decreased significantly in a dose-dependent manner. Scale bar = 50 *μ*m. ^*∗∗*^*P* < 0.01*ν*s. the control group.

The results of the MTT assay showed that the absorbance values of SHI-1 cells treated with Rk3 60, 120, and 180 mol/L were 0.73 ± 0.07, 0.51 ± 0.05, and 0.39 ± 0.04, respectively, which were significantly lower than the 0.95 ± 0.05 of the control group (*P* < 0.01). The inhibition rates of Rk3 were 24.3%, 46.7%, and 59.7%, in a dose-dependent manner, with 136.02 ± 15.03 mol/L of the IC_50_ in SHI-1 cells in vitro, as shown in Figure 1(a). The results of the colony formation assay showed that the colony numbers were 102.6 ± 8.7/10^3^, 78.4 ± 7.1/10^3^, and 53.2 ± 7.3/10^3^ cells after being treated by Rk3 at 60, 120, and 180 mol/L for 7 days, which were significantly lower than the 164.6 ± 7.1/10^3^ cells of the control group (*P* < 0.01). The inhibition rates were 37.4%, 52.3%, and 67.7%, respectively, in a dose-dependent manner with 119.7 ± 7.2 mol/L of the IC_50_, as shown in Figure 1(b).

### 3.2. Rk3 Inhibited the Migration and Invasion of SHI-1 Cells

**Figure 2:**
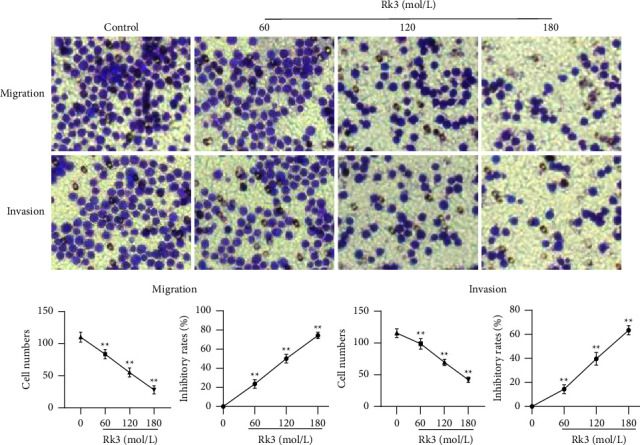
The inhibition effect of Rk3 on migration and invasion in SHI-1 cells. ($\bar{x}\pm s$, *n* = 5) Transwell assay showed that the number of cell migrations and invasions were significantly decreased in Rk3 treated SHI-1 cells at the concentration of 60, 120, and 180 mol/L. ^*∗∗*^*P* < 0.01 vs. the control group.

The inhibition effect of Rk3 on migration and invasion were observed by the Transwell assay. The number of cell migrations in Rk3 groups of 60, 120, and 180 mol/L were 83.6 ± 6.4, 55.8 ± 6.3, and 28.4 ± 5.7, respectively, which were significantly lower than the 110 ± 6.0 in the control group (*P* < 0.01). The inhibition rates of Rk3 on the migration of SHI-1 cells were from 23.7% to 74.4%. The similar results were also observed in the invasion assay, the number of cell invasions in the 60, 120, and 180 mol/L groups were 98.7 ± 7.4, 69.5 ± 4.3, and 42.2 ± 3.7, respectively, which was lower than the 115.5 ± 6.2 in the control group (*P* < 0.01). The inhibition rates of Rk3 on the invasion of SHI-1 cells were from 14.5% to 63.4%, as shown in Figure 2.

### 3.3. The Correlation between the Expression of miR-3677-5p, CXCL12, and CXCR4 and EMI in M5 Patients

**Figure 3:**
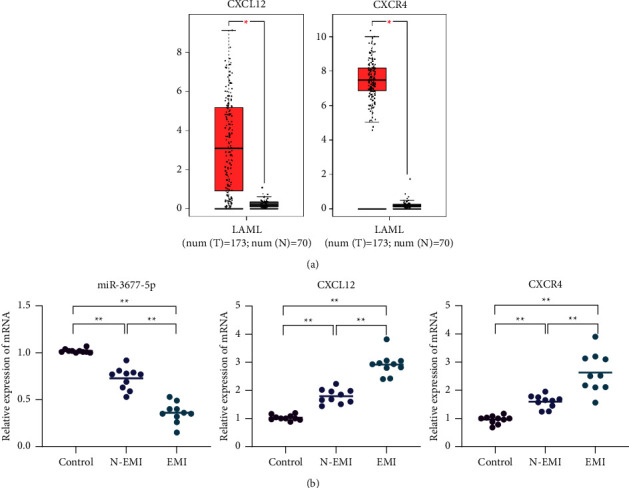
The expression levels of miR-3677-5p, CXCL12, and CXCR4 in leukemia cells. (a) The GEPIA website showed that the expression levels of CXCL12 and CXCR4 were significantly increased in AML patients compared with healthy people. (b) RT-qPCR showed that the expression of miR-3677-5p in the EMI group was significantly lower than that in both the N-EMI group and the normal control group. However, the mRNA expression of CXCL12 and CXCR4 was higher than those of the N-EMI group and the normal control group. ^*∗∗*^*P* < 0.01*ν*s. the control group.

The results of GEPIA website showed that the expression levels of CXCL12 and CXCR4 in AML patients were significantly higher than those in healthy people (*P* < 0.05), as shown in Figure 3(a). The relative expression level of miR-3677-5p in AML-M5 patients was significantly lower than that in normal control group (*P* < 0.01). Meanwhile, the relative expression level of miR-3677-5p in EMI group was 0.36 ± 0.10, which was lower than the 0.73 ± 0.11 in N-EMI group (*P* < 0.01). The mRNA relative expression levels of CXCL12 and CXCR4 in AML-M5 patients was significantly higher than those in the normal control group (*P* < 0.01). Meanwhile, the mRNA relative expression levels of CXCL12 and CXCR4 in the EMI group were 2.92 ± 0.37 and 2.63 ± 0.66, respectively, which were significantly higher than 1.79 ± 0.24 and 1.60 ± 0.22 in N-EMI group (*P* < 0.01), as shown in Figure 3(b). The leukemia cells in the EMI group showed abnormally low expression of miR-3677-5p but high expression of CXCL12 and CXCR4 mRNA, indicating that the miR-3677-5p/CXCL12 axis may be closely related to the occurrence of EMI in monocytic leukemia. Meanwhile, clinical data showed that the proportion of bone marrow blasts in patients of the EMI group was 68.4 ± 12.5%, significantly higher than the 46.5 ± 16.7% in the N-EMI group. Furthermore, the patients of the EMI group lacked sensitivity to chemotherapy, and the remission rate after chemotherapy was low, as shown in Table 1.

### 3.4. The Expression Levels of miR-3677-5p, CXCL12, and CXCR4 Either Lentivirus Transfection or AMD3100 Intervention

**Figure 4:**
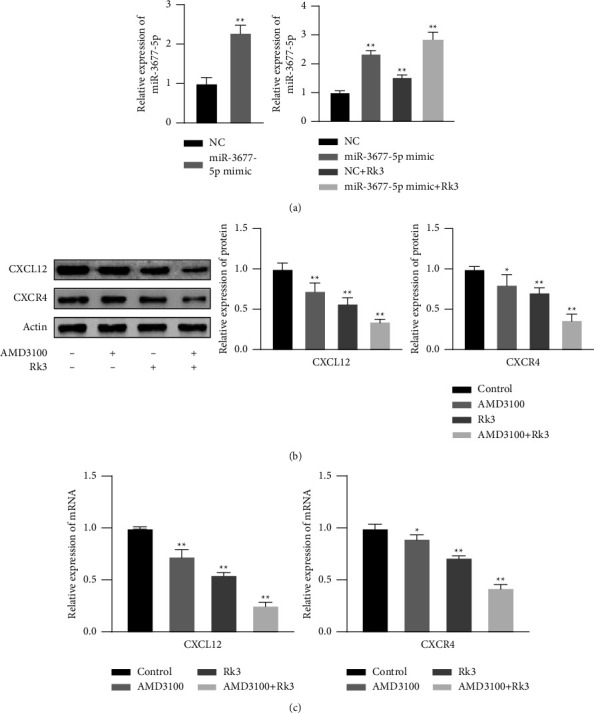
The expression levels of miR-3677-5p, CXCL12, and CXCR4 either lentivirus transfection or AMD3100 intervention in SHI-1 cells. ($\bar{x}\pm s$, *n* = 3) (a) The expression of miR-3677-5p detected by RT-qPCR in NC, miR-3677-5p mimic, NC + Rk3, and miR-3677-5p mimic + Rk3 groups. (b) The expression of CXCL12 and CXCR4 protein detected by Western blot in both the AMD3100 and Rk3 alone or combination groups; (c) The mRNA expression levels of CXCL12 and CXCR4 analyzed by RT-qPCR were consistent with Western blot results. ^*∗*^*P* < 0.05, ^*∗∗*^*P* < 0.01 vs. the control group.

RT-qPCR showed that the relative expression levels of miR-3677-5p in NC and miR-3677-5p mimic groups were 1.00 ± 0.12 and 2.29 ± 0.15, respectively. The miR-3677-5p mimic group showed significantly higher expression of miR-3677-5p than that of the NC group (*P* < 0.01), indicated that lentivirus miR-3677-5p mimic was successfully transfected, as shown in Figure 4(a). The relative expression levels of miR-3677-5p in miR-3677-5p mimic group, NC + Rk3 120 mol/L group, and miR-3677-5p mimic + Rk3 120 mol/L group were 2.36 ± 0.07, 1.54 ± 0.06, and 2.84 ± 0.21, respectively, all of them were significantly higher than 1.00 ± 0.03 in the control group (*P* < 0.01), as shown in Figure 4(a). SHI-1 cells were treated by both AMD3100 2.5 *μ*M and Rk3 120 mol/L alone or combination, the expression of CXCL12 in each group were 0.73 ± 0.08, 0.57 ± 0.06, and 0.35 ± 0.02, respectively, which were significantly lower than 1.00 ± 0.06 in the control group (*P* < 0.01). Meanwhile, the expression of CXCR4 in each group were 0.81 ± 0.10, 0.71 ± 0.05, and 0.37 ± 0.06, which were significantly lower than 1.00 ± 0.03 in the control group (*P* < 0.05 or 0.01), as shown in Figure 4(b). The RT-qPCR were consistent with Western blot results, the mRNA expression levels of CXCL12 and CXCR4 in each group above were significantly lower than those in the control group (*P* < 0.05 or 0.01), as shown in Figure 4(c).

### 3.5. Overexpression of miR-3677-5p Could Inhibit the Proliferation, Migration, and Invasion

**Figure 5:**
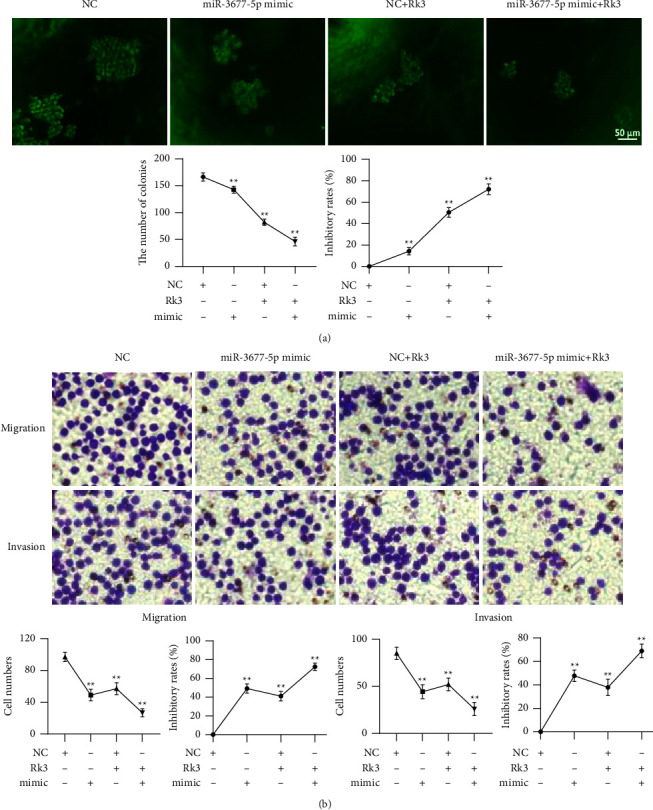
Overexpressed miR-3677-5p could inhibit proliferation, migration, and invasion of SHI-1 cells. ($\bar{x}\pm s$, *n* = 5) (a) Colony formation assay showed that overexpressed miR-3677-5p effectively inhibited the proliferation of SHI-1 cells; (b) Transwell assay showed that overexpressed miR-3677-5p also significantly inhibited migration and invasion. ^*∗∗*^*P* < 0.01 vs. the control group.

The colony numbers in miR-3677-5p mimic, NC + Rk3 120 mol/L, and miR-3677-5p mimic + Rk3 120 mol/L groups were 142.8 ± 5.8/10^3^, 82.2 ± 5.3/10^3^, and 46.4 ± 7.1/10^3^, respectively, which were significantly lower than the 166.4 ± 6.7/10^3^ in the control group (*P* < 0.01), and the inhibition rates were 14.1%, 50.5%, and 72.0% (*P* < 0.01), respectively, as shown in Figure 5(a). The migration assay showed that the number of cell migrations in the above three groups were 49.8 ± 6.4, 57.4 ± 6.7, and 26.7 ± 4.5, respectively, which were also significantly lower than 97.6 ± 5.1 in the control group (*P* < 0.01), and the inhibition rates were 49.3%, 41.2%, and 72.4%, respectively. The number of cell invasions in the above three groups were 44.3 ± 6.7, 52.2 ± 6.0, and 26.6 ± 6.2, respectively, which were also significantly lower than the 85.6 ± 6.0 in the control group (*P* < 0.01), and the inhibition rates were 47.8%, 38.0%, and 68.9%, respectively, as shown in Figure 5(b).

### 3.6. CXCL12 Is the Downstream Target Gene of miR-3677-5p

**Figure 6:**
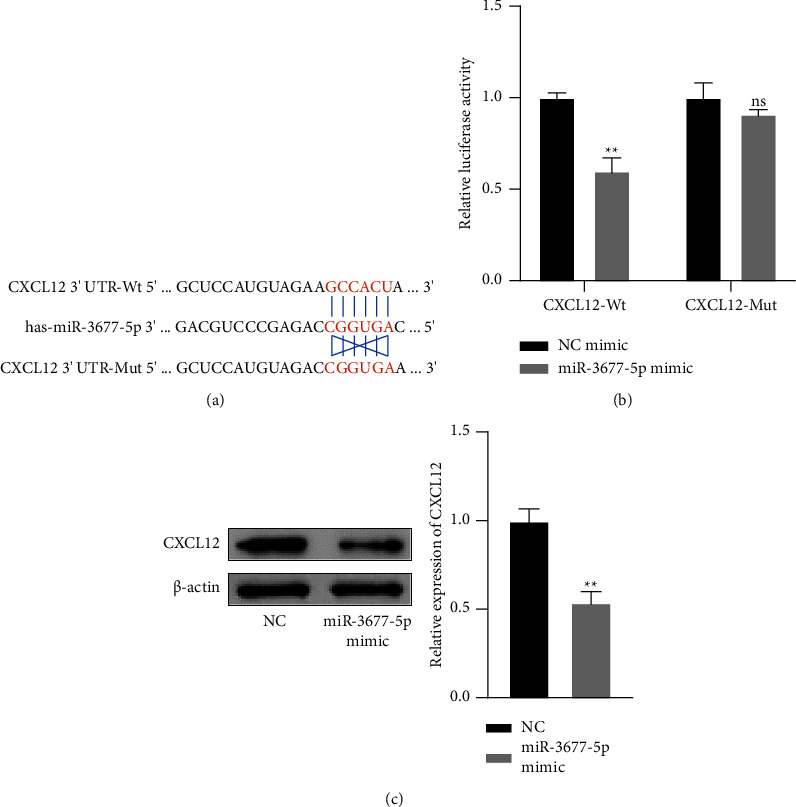
CXCL12 is the downstream target gene of miR-3677-5p. ($\bar{x}\pm s$, *n* = 3) (a) The base pairing sequence between miR-3677-5p and CXCL12 3′ UTR was predicted by TargetScan database, the dual luciferase vector with CXCL12 3′ UTR-Wt and CXCL12 3′ UTR-Mut was inserted into the plasmids. (b) Luciferase assay verified the targeting relationship between miR-3677-5p and CXCL12. (c) The expression of CXCL12 in the NC and miR-3677-5p mimic groups. ^*∗∗*^*P* < 0.01 vs. the control group.

The specific binding sites between miR-3677-5p and CXCL12 were predicted by using biological information database, as shown in Figure 6(a). Furthermore, luciferase assay showed that CXCL12 3′ UTR-Wt and miR-3677-5p mimic were co-transfected into 293T cells, and the luciferase activity was significantly decreased in compared with NC mimic group (*P* < 0.01). CXCL12 3′ UTR-Mut and miR-3677-5p mimic were co-transfected into 293T cells, but there was no significant difference between the two groups, as shown in Figure 6(b). The Western blot results showed that overexpression of miR-3677-5p significantly inhibited the expression level of CXCL12 protein in SHI-1 cells (*P* < 0.01), as shown in Figure 6(c).

### 3.7. Intervening CXCL12 Could Inhibit Proliferation, Migration, and Invasion

**Figure 7:**
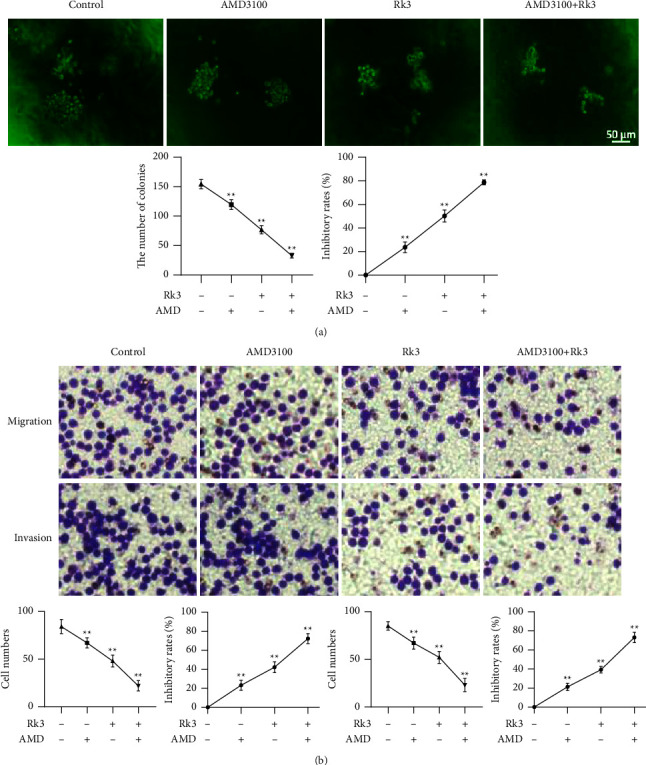
Intervening CXCL12 could inhibit proliferation, migration, and invasion of SHI-1 cells. ($\bar{x}\pm s$, *n* = 5) (a) Colony formation assay showed that both the AMD3100 and Rk3 could inhibit the proliferation of SHI-1 cells either alone or combination. (b) Transwell assay showed that the number of cell migrations and invasions of AMD3100 and Rk3 either alone or combination were significantly lower than those of the control group. ^*∗∗*^*P* < 0.01 vs. the control group.

The colony forming numbers of AMD3100, Rk3 120 mol/L, and AMD3100 + Rk3 120 mol/L groups were 119.6 ± 7.5/10^3^, 76.8 ± 6.3/10^3^, and 32.8 ± 3.5/10^3^, respectively, which was significantly lower than 154.4 ± 7.0/10^3^ in the control group (*P* < 0.01), and the inhibitory rates were 22.4%, 50.2%, and 78.8% (*P* < 0.01), respectively, as shown in Figure 7(a). The Transwell assay showed that the number of cell migrations in the abovementioned three groups were 67.2 ± 4.8, 48.0 ± 5.5, and 22.4 ± 4.8, respectively, which was significantly lower than 84.3 ± 6.6 in the control group (*P* < 0.01), and the inhibition rates were 20.6%, 42.2%, and 72.2%, respectively. The number of cell invasions in the above three groups were 67.8 ± 5.6, 52.0 ± 5.6, and 23.8 ± 6.2, respectively, which were also significantly lower than 85.0 ± 4.0 in the control group (*P* < 0.01), and the inhibition rates were 21.2%, 39.1%, and 73.0%, respectively, as shown in Figure 7(b).

### 3.8. The Expression of miR-3677-5p, CXCL12, and CXCR4 in SHI-1 Cells after Treatment of Rk3

**Figure 8:**
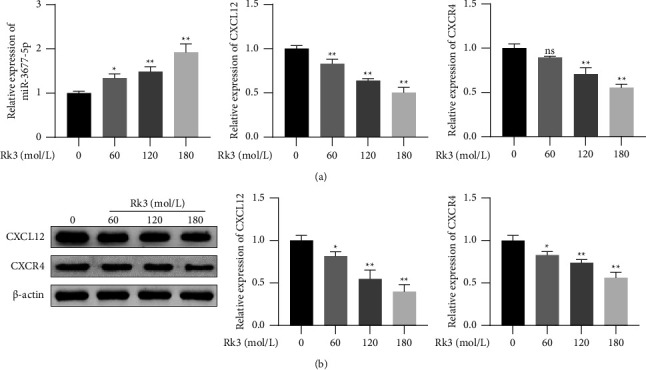
The expression of miR-3677-5p, CXCL12, and CXCR4 in SHI-1 cells after treatment of Rk3. ($\bar{x}\pm s$, *n* = 3) SHI-1 cells were treated with Rk3 at 60, 120, and 180 mol/L for 48 h. (a) RT-qPCR showed that Rk3 upregulated the expression of miR-3677-5p, while downregulated the mRNA expression of CXCL12 and CXCR4. (b) Western blot analysis showed that Rk3 significantly downregulated the expression of CXCL12 and CXCR4 proteins. ^*∗*^*P* < 0.05, ^*∗∗*^*P* < 0.01 vs. the control group.

RT-qPCR showed that the expression level of miR-3677-5p in SHI-1 cells treated by Rk3 at 60, 120, and 180 mol/L was significantly higher than that in the control group, but the mRNA expression level of CXCL12 was significantly lower than that in the control group (*P* < 0.05 or 0.01), as shown in Figure 8(a). The expression level of CXCR4 in the Rk3 120 and 180 mol/L groups was significantly lower than that in the control group (*P* < 0.01). The result of the Western blot was consistent with that of the RT-qPCR above. The protein levels of both CXCL12 and CXCR4 in Rk3 treated cells were significantly lower than those in the control group (*P* < 0.05), as shown in Figure 8(b).

### 3.9. Rk3 Regulated the Expression of EMI-Related Functional Proteins

**Figure 9:**
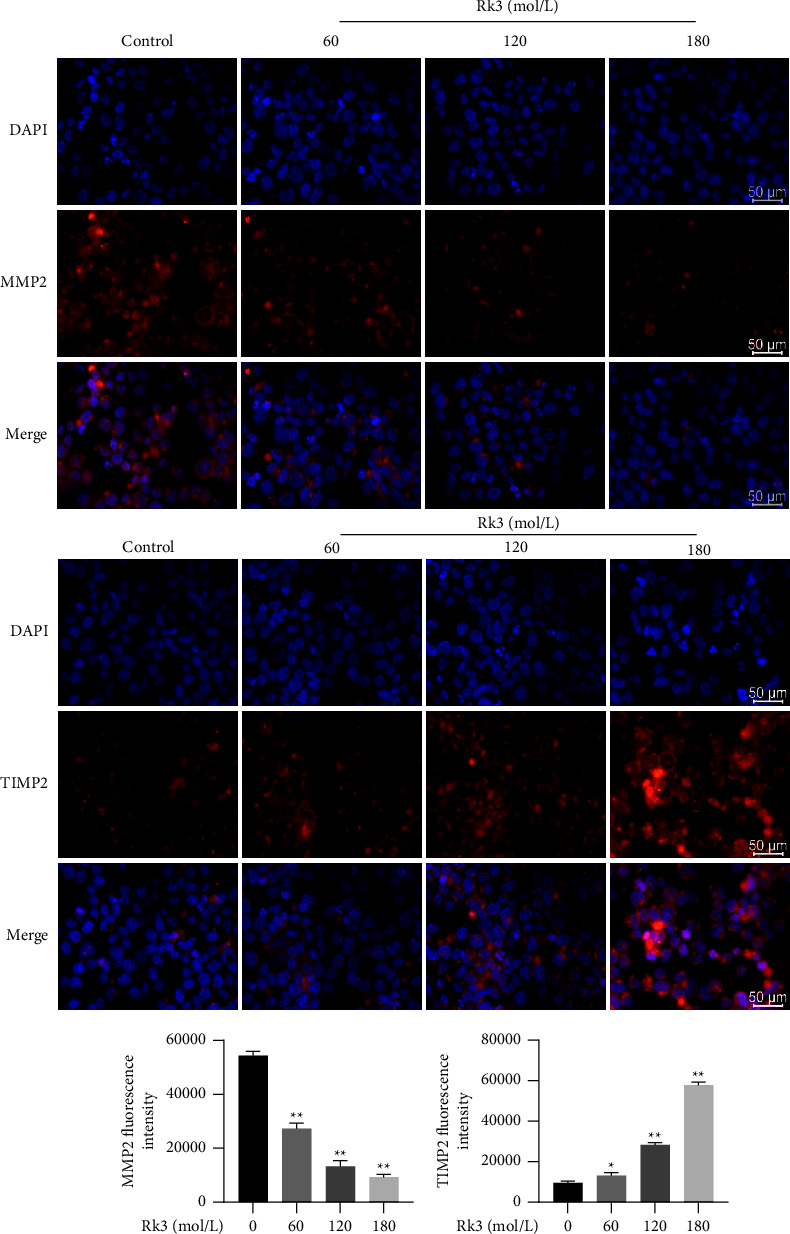
Rk3 regulated the expression of EMI-related functional proteins. The SHI-1 cells treated by Rk3 showed the fluorescence intensity of MMP2 protein was significantly weakened, while the fluorescence intensity of TIMP2 was significantly enhanced. Scale bar = 50 *μ*m.

The results of immunofluorescence showed the fluorescence intensity of MMP2 protein related to promoting migration and invasion, which was significantly weakened in Rk3 treated SHI-1 cells, while the fluorescence intensity of TIMP2 related to inhibiting migration and invasion was significantly enhanced. The results indicated that Rk3 could not only directly inhibit the expression of MMP2 protein but also upregulate the expression of TIMP2 protein, so as to enhance the inhibitory effect of Rk3 on MMP2 protein, as shown in Figure 9.

### 3.10. The Effect of miR-3677-5p-Regulating CXCL12 on the Expression of EMI-Related Functional Proteins

**Figure 10:**
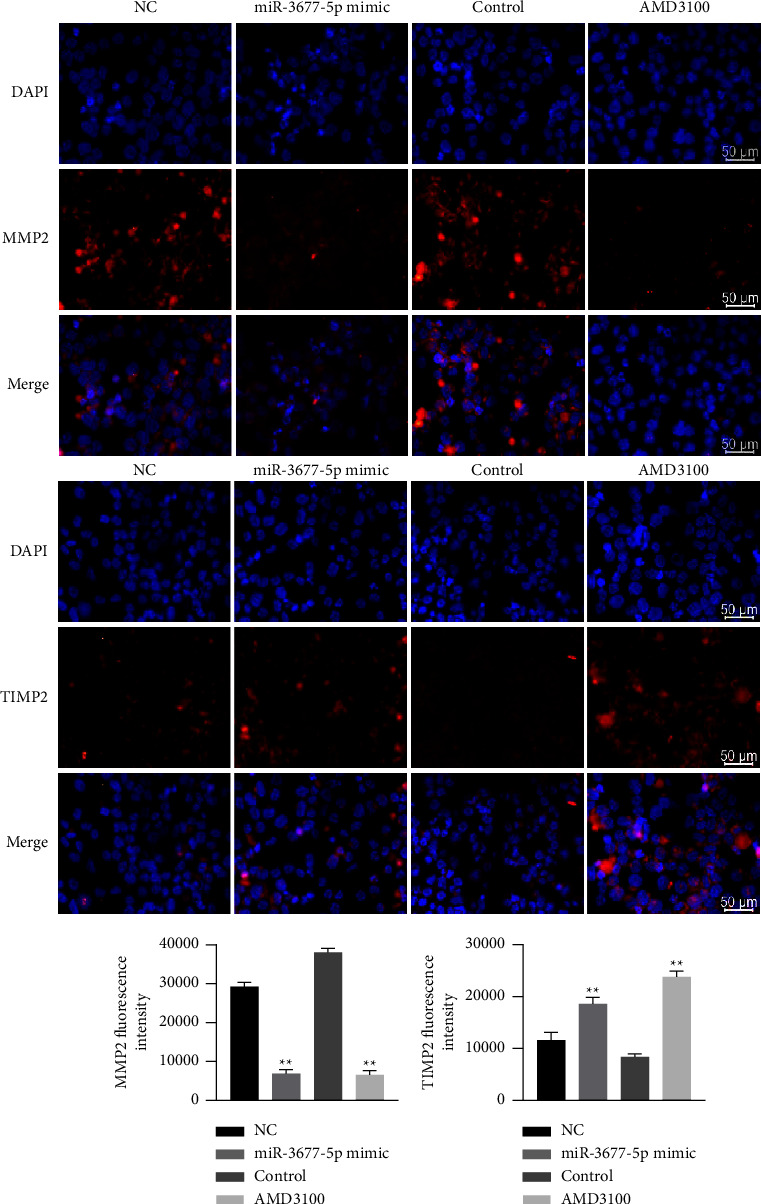
The effect of miR-3677-5p-regulating CXCL12 on the expression of EMI-related functional proteins. The immunofluorescence results showed that the fluorescence intensity of MMP2 and TIMP2 in each group. Scale bar = 50 *μ*m.

The immunofluorescence results showed that compared with the miR-NC group and control group, the fluorescence intensity of MMP2 in the miR-3677-5p mimic group and AMD3100 group was significantly weakened, while the fluorescence intensity of TIMP2 was significantly enhanced, as shown in Figure 10.

## 4. Discussion

Acute monocytic leukemia belongs to type M5 of AML, classified by FAB, with two different morphological subtypes (M5a and M5b), characterized by abnormal proliferation of immature monocytes in peripheral blood and bone marrow, eventually resulting in ineffective hematopoiesis and bone marrow failure. Clinical complications are anemia, infection, bleeding, and so on. M5 patients often have EMI in the liver, spleen, lymph nodes, skin, and gums. Infiltration of the central nervous system occurs mostly in the spinal cord membrane, brain parenchyma, and spinal cord. That is, leukemia cells directly spread or hematogenous transfer into the central nervous system, which is one of the important factors leading to recurrence and death [24]. The mechanism of EMI is a complex process of multistep and multifactor regulation, involving multiple factors of activation or inhibition. First, leukemia cells escape from bone marrow to peripheral blood, undergo chemotaxis, and adhere to the vascular endothelium through adhesion molecules, migrate and degrade the extracellular matrix, then survive and proliferate in extramedullary tissues, and finally, form infiltrating lesions [25]. In terms of cytogenetic factors, the occurrence of EMI is usually associated with the alterations in chromosome 8 alteration, FLT3-ITD and NPM1 mutations [26]. Most patients with EMI had a low remission rate and a short overall survival time after chemotherapy [27]. Cytotoxic chemotherapy drugs, targeted therapies, and immunotherapies cannot prevent and cure EMI, so it is necessary to research and develop a safe and effective new Chinese medicine as an adjuvant therapy for anti-EMI of leukemia.

Ginsenoside Rk3 is isolated from total saponins of panax ginseng, it has been reported that Rk3 is effective to inhibit the proliferation and induce apoptosis of tumor cells in esophageal cancer, liver cancer, nonsmall cell lung cancer, and other tumors. For instance, Rk3 could decrease the expression of inflammatory factors, arrest cell cycle, promote apoptosis of hepatocellular carcinoma cells, and therefore, inhibit the occurrence and development of hepatocellular carcinoma by targeting the LPS-TLR4 signaling pathway [28]. Rk3 could downregulate the expression of cyclin D1 and CDK4, upregulate the expression of P21 protein, and inhibit the proliferation and colony formation of lung cancer cells [29]. Consistently, in this study, we observed Rk3 was effective to inhibit the proliferation, migration, and invasion of SHI-1 cells, and regulate miR-3677-5p/CXCL12 axis and EMI-related functional proteins.

Some studies indicated that targeted upregulation of miR-3677 expression level would inhibit the proliferation and migration of human osteosarcoma cells [18]. Our study showed that the primary leukemia cells of patients in the EMI group showed abnormally low expression of miR-3677-5p. Meanwhile, these patients with EMI were not sensitive to chemotherapy with a low remission rate. After miR-3677-5p mimic was transfected, the expression of miR-3677-5p in SHI-1 cells increased, and the proliferation ability decreased significantly. The Transwell experiment found that overexpression of miR-3677-5p could inhibit the migration and invasion in SHI-1 cells and regulate EMI-related functional proteins. Our results showed that Rk3 upregulated the expression of miR-3677-5p. The abovementioned results all indicated that miR-3677-5p could inhibit the malignant behavioral activities of monocytic leukemia cells and can be used as a specific target for targeted therapy of monocytic leukemia.

The literature reported that CXCL12/CXCR4 axis was extensively involved in the migration and invasion of colorectal cancer and other cancers [30, 31]. Adhesion molecules were identified as key proteins in relation to tumor cell invasion, and CXCL12 could also upregulate the expression of adhesion molecules such as VLA-4. The results of the GEPIA website showed that the expression levels of CXCL12 and CXCR4 in AML patients were significantly higher than those in healthy people. Meanwhile, our results showed that patients' leukemia cells in the EMI group showed abnormally high expression of CXCL12 and CXCR4 mRNA. In combination with the clinical situation, these patients with EMI were not sensitive to chemotherapy with a low remission rate. After interfering with the expression of CXCL12, the proliferation, migration, and invasion of SHI-1 cells were inhibited. In addition, TargetScan database found that miR-3677-5p and CXCL12 have specific binding sites, it have been confirmed that miR-3677-5p could directly bind to the 3′ UTR of CXCL12 and inhibit its activity, by means of dual luciferase reporter gene assay. The experimental results above indicate that the miR-3677-5p/CXCL12 axis may be closely related to the occurrence of EMI in monocytic leukemia. Therefore, miR-3677-5p and CXCL12 may be used as indicators of EMI and poor prognosis in monocytic leukemia. In view of the small number of cases in this study, the cases need to be expanded for further verification.

EMI and N-EMI have the same pathogenesis, but they are two manifestations of AML. The reason for the difference between EMI and N-EMI was the heterogeneity and complexity of leukemia cells. That is, leukemia cells in the EMI group had a higher degree of malignancy and possessed extremely strong proliferation potential, so the proportion of bone marrow blasts in patients with the EMI group was significantly higher than that in the N-EMI group. The potency of both extramedullary migration and invasion was very strong for leukemia cells in the EMI group, which is one of the important factors for poor prognosis.

The MMPs are zinc-dependent proteolytic enzymes of the extracellular matrix (ECM) [32]. As a member of MMPs, MMP2 is involved in the invasion of a variety of tumor cells by digesting the extracellular matrix barrier [33–37]. Excessive secretion of MMP2 by leukemia cells would increase the permeability of the blood-brain barrier via disrupting the tight junction proteins, leading to enhance the invasion of leukemia cells into the central nervous system [38]. In addition, the TIMPs family is a tissue inhibitor of matrix metalloproteinases, among them, TIMP2 is capable of specifically suppressing the expression and activity of MMP2. After overexpression of miR-3677-5p or interference with CXCL12 expression, the fluorescence intensity of MMP2 weakened, and the fluorescence intensity of TIMP2 enhanced. Our results showed that Rk3 downregulated the expression of MMP2, but upregulated the expression of TIMP2. It shows that the high expression of miR-3677-5p and the low expression of CXCL12 can significantly inhibit the cell EMI process, reducing the migration and invasion.

In summary, our results indicated that the ginsenoside Rk3 is effective to inhibit proliferation, migration, and invasion by targeting miR-3677-5p/CXCL12 axis in SHI-1 cells, it provided experimental evidence for the role of Rk3 on antimonocytic leukemia, but to elucidate the exact mechanism remains to be further explored.

## Figures and Tables

**Table 1:** The patients' clinical manifestation and general data.

Family name	Group case no.	FAB classification	BM blast cells (%)	Extramedullar infiltrating organ	Chemotherapy regimens	Chemotherapy response	miR-3677-5p expression	CXCL12 expression	CXCR4 expression
Chen	EMI-1	M5a	49	Lung	DAV	NR	0.4	2.4	3.9
Tao	EMI-2	M5a	68	Pancrea, spleen, and neck lymph gland	DAV	NR	0.5	2.9	3.1
Chen	EMI-3	M5b	60	Lymph gland of whole body	DA	CR	0.2	2.8	2.2
Ping	EMI-4	M5b	80	Spleen and neck lymph gland	DA	NR	0.3	3.8	2.1
Sun	EMI-5	M5a	87	Spleen and groin lymph gland	DA	NR	0.4	2.9	2.5
Guo	EMI-6	M5a	86	Neck lymph gland	DA	CR	0.4	3.1	2.1
Du	EMI-7	M5b	73	Lung	DAV	NR	0.4	2.8	3.2
Gong	EMI-8	M5b	53	Neck lymph gland	DA	PR	0.3	3.1	3.1
Qi	EMI-9	M5a	67	Groin lymph gland	DAV	NR	0.5	3.0	1.6
Lou	EMI-10	M5a	61	Spleen and neck lymph gland	DAV	CR	0.4	2.4	2.5
Mao	N-EMI-1	M5b	26	—	DA	CR	0.8	1.7	1.5
Yuan	N-EMI-2	M5a	37	—	VA	NR	0.6	1.5	2.0
Rao	N-EMI-3	M5b	53	—	VA	PR	0.8	1.6	1.6
Zhou	N-EMI-4	M5a	75	—	VA	PR	0.6	2.2	1.6
Wang	N-EMI-5	M5a	36	—	DA	PR	0.9	2.0	1.3
Cheng	N-EMI-6	M5b	75	—	DAV	PR	0.8	2.0	1.7
Wang	N-EMI-7	M5b	42	—	DAV	PR	0.8	1.9	1.8
Zhang	N-EMI-8	M5a	34	—	DAV	CR	0.8	1.4	1.8
Cheng	N-EMI-9	M5b	31	—	VA	CR	0.7	1.7	1.7
Wang	N-EMI-10	M5a	56	—	DAV	CR	0.5	2.0	1.2

Chemotherapy: (1) DAV. DNR + Ara-C + venetoclax; (2) DA. DNR + Ara-C; (3) VA. venetoclax + Ara-C.

## Data Availability

All data generated or analyzed during this study are included in this article.
